# Supplementary material for: Metabolic reprogramming and altered cell envelope characteristics in a pentose phosphate pathway mutant increases MRSA resistance to β-lactam antibiotics
Source: PLoS Pathog. 2023 Jul 24;19(7):e1011536. doi: 10.1371/journal.ppat.1011536 (PMC10399904; doi:10.1371/journal.ppat.1011536)
Supplement: S1 Table — (DOCX) [file ppat.1011536.s001.docx]

**S1 Table.** Oxacillin minimum inhibitory concentrations (MICs, µg/ml) of JE2 and *pgl* grown in CDMG or MHB 2% NaCl supplemented with 10-70% (v/v) human serum.

| **Strain** | **Oxacillin MICs** | | | |
| --- | --- | --- | --- | --- |
|  | 10% serum | 25% serum | 50% serum | 70% serum |
|  | CDMG | | | |
| **JE2** | 4 | 4-8 | 8-32 | 32 |
| ***pgl*** | 256 | 512 | 512 | >512 |
|  | MHB 2% NaCl | | | |
| **JE2** | 64 | 64 | 64 | 32-64 |
| ***pgl*** | 256 | 256 | 256 | 256 |
