# Supplementary material for: Metabolic reprogramming and altered cell envelope characteristics in a pentose phosphate pathway mutant increases MRSA resistance to β-lactam antibiotics
Source: PLoS Pathog. 2023 Jul 24;19(7):e1011536. doi: 10.1371/journal.ppat.1011536 (PMC10399904; doi:10.1371/journal.ppat.1011536)
Supplement: S2 Table — (DOCX) [file ppat.1011536.s002.docx]

**S2 Table.** Bacterial strains and plasmids used in this study

| **Strains/plasmids** | **Relevant Details** |
| --- | --- |
|  | |
| ***Staphylococcus aureus* strains** | |
| JE2 | USA300 cured of p01 & p03. Parent strain from the Nebraska Transposon Mutant Library (NTML). [1] |
| BH1CC | MRSA clinical isolate; SCC*mec* type II; CC8 [2] |
| 8325-4 | NCTC 8325 derivative cured of prophages [3], MSSA, CC8. |
| RN4220 | Restriction deficient derivative of *S. aureus* 8325 [3] |
| NE202 (*pgl*) | JE2 *pgl*::Erm^r^ (SAUSA300_1902). Erm^r^. [1] |
| *pgl*::Km^r^ | JE2 *pgl*::Km, Km^r^ |
| *pgl*R1 | JE2 Δ*pgl*, VraG Gln_394_STOP |
| NE202 pLI50_*pgl* (*pgl*_comp_) | NE202 pLI50_*pgl* Erm^r^, Cm^r^ |
| NE952 (*gntP*) | JE2 *gntP*. Erm^r^. [1] |
| NE1124 (*gntK*) | JE2 *gntK*. Erm^r^. [1] |
| NE569 (*sucC*) | JE2 *sucC*. Erm^r^. [1] |
| NE547 (*sucA*) | JE2 *sucA*. Erm^r^. [1] |
| NE76 (*leuB*) | JE2 *leuB*. Erm^r^. [1] |
| NE239 (*putA*) | JE2 *putA*. Erm^r^. [1] |
| NE1518(*gudB*) | JE2 *gudB*. Erm^r^. [1] |
| NE70 (*vraG*) | JE2 *vraG*. Erm^r^. [1] |
| NE645 (*vraF*) | JE2 *vraF*. Erm^r^. [1] |
| NE481 (*graR*) | JE2 *graR*. Erm^r^. [1] |
| NE1868 (*mecA*) | JE2 *mecA*. Erm^r^. [1] |
| NE626 *(sdhA)* | JE2 *sdhA*. Erm^r^. [1] |
| NE942 (*tarS*) | JE2 *tarS*. Erm^r^. [1] |
| NE611 (*tarM*) | JE2 *tarM*. Erm^r^. [1] |
| JE2 *pgl*::Erm^r^ | JE2 transductant. *pgl*::Erm^r^. This study. |
| JE2 *gntP*::Erm^r^ | JE2 transductant. *gntP*::Erm^r^. This study. |
| JE2 *gntK*::Erm^r^ | JE2 transductant. *gntK*::Erm^r^. This study. |
| *pgl/gntP* | Km^r^, Erm^r^. This study. |
| *pgl/gntK* | Km^r^, Erm^r^. This study. |
| *pgl/sucC* | Km^r^, Erm^r^. This study. |
| *pgl/sucA* | Km^r^, Erm^r^. This study. |
| *pgl/leuB* | Km^r^, Erm^r^. This study. |
| *pgl/putA* | Km^r^, Erm^r^. This study. |
| *pgl/gudB* | Km^r^, Erm^r^. This study. |
| *pgl/vraG* | Km^r^, Erm^r^. This study. |
| *pgl/vraF* | Km^r^, Erm^r^. This study. |
| *pgl/graR* | Km^r^, Erm^r^. This study. |
| *pgl/thrC* | Km^r^, Erm^r^. This study. |
| *pgl/mecA* | Km^r^, Erm^r^. This study. |
| *pgl/sdhA* | Km^r^, Erm^r^. This study. |
| *pgl/putA_Spec_* | Km^r^, Spec^r^. This study. |
| *pgl/putA/vraG* | Km^r^, Spec^r^, Erm^r^. This study. |
| *pgl/tarS* | Km^r^, Erm^r^. This study. |
| *pgl/tarM* | Km^r^, Erm^r^. This study. |
| LAC* (ANG1575) | LAC*; Erm sensitive CA-MRSA LAC strain (AH1263)[4] |
| *ltaS/gdpP* (ANG2434) | Erm^r^, US3 pass4: LAC*Δ*ltaS::erm* *gdpP* suppressor [5] |
| *tagO* (ANG4759) | LAC* ∆*tagO* [6] |
|  |  |
| ***Escherichia coli* strains** |  |
| *E. coli* TOP10 | (F- *mcrA* Δ(*mrr*-*hsdRMS*-*mcrBC*) φ80*lacZ*ΔM15 Δ*lacX*74 *nupG* *recA1* *araD139* Δ(araleu)7697 *galE15* *galK16* *rpsL*(StrR ) *endA1* λ (Invitrogen) |
| *E. coli* HST08 | F- ,*endA1*, *supE44*,*thi*-1,*recA1*,*relA1*,*gyrA96*,*phoA*, Φ80d*lacZ*ΔM15, Δ (*lacZYA -argF* ) U169, Δ (*mrr* -*hsdRMS* - *mcrBC* ), *ΔmcrA*,λ– (Takara Bio) |
| HST08 pLI50_*pgl* | *E. coli* HST08 carrying pLI50_*pgl*. Amp^r^. This study. |
| IM08B | SA08BΩPN25-*hsdS* (CC8-1) (SAUSA300_0406) of NRS384 integrated between the *essQ* and *cspB* genes [7] |
| **Plasmids** |  |
| pDrive | *E. coli* cloning vector (Qiagen) |
| pDrive_*pgl* | pDrive carrying *pgl* from JE2. This study. |
| pLI50 | *E. coli* (Amp^r^)*-Staphylococcus* (Cm^r^)shuttle vector [8] |
| pLI50_*pgl* | pLI50 carrying *pgl* from JE2. *E. coli* (Amp^r^)*-Staphylococcus* (Cm^r^). This study. |
| pTNT | pJB38 with homologous DNA to *bursa aurealis* [9] |
| pKAN | pTNT with *aphA-3* [9] |
| pSPC | pTNT with *aad9* [9] |

**References:**

1. Fey PD, Endres JL, Yajjala VK, Widhelm TJ, Boissy RJ, Bose JL, et al. A genetic resource for rapid and comprehensive phenotype screening of nonessential Staphylococcus aureus genes. MBio. 2013;4(1):e00537-12.

2. O'Neill E, Pozzi C, Houston P, Smyth D, Humphreys H, Robinson DA, et al. Association between methicillin susceptibility and biofilm regulation in Staphylococcus aureus isolates from device-related infections. J Clin Microbiol. 2007;45(5):1379-88.

3. Horsburgh MJ, Aish JL, White IJ, Shaw L, Lithgow JK, Foster SJ. sigmaB modulates virulence determinant expression and stress resistance: characterization of a functional rsbU strain derived from Staphylococcus aureus 8325-4. J Bacteriol. 2002;184(19):5457-67.

4. Boles BR, Thoendel M, Roth AJ, Horswill AR. Identification of genes involved in polysaccharide-independent Staphylococcus aureus biofilm formation. PLoS One. 2010;5(4):e10146.

5. Corrigan RM, Abbott JC, Burhenne H, Kaever V, Gründling A. c-di-AMP is a new second messenger in Staphylococcus aureus with a role in controlling cell size and envelope stress. PLoS Pathog. 2011;7(9):e1002217.

6. Schuster CF, Wiedemann DM, Kirsebom FCM, Santiago M, Walker S, Grundling A. High-throughput transposon sequencing highlights the cell wall as an important barrier for osmotic stress in methicillin resistant *Staphylococcus aureus* and underlines a tailored response to different osmotic stressors. Mol Microbiol. 2020;113(4):699-717.

7. Monk IR, Tree JJ, Howden BP, Stinear TP, Foster TJ. Complete Bypass of Restriction Systems for Major Staphylococcus aureus Lineages. Mbio. 2015;6(3).

8. Lee CY, Buranen SL, Ye ZH. Construction of single-copy integration vectors for Staphylococcus aureus. Gene. 1991;103(1):101-5.

9. Bose JL, Fey PD, Bayles KW. Genetic tools to enhance the study of gene function and regulation in Staphylococcus aureus. Appl Environ Microbiol. 2013;79(7):2218-24.
