# Supplementary material for: Metabolic reprogramming and altered cell envelope characteristics in a pentose phosphate pathway mutant increases MRSA resistance to β-lactam antibiotics
Source: PLoS Pathog. 2023 Jul 24;19(7):e1011536. doi: 10.1371/journal.ppat.1011536 (PMC10399904; doi:10.1371/journal.ppat.1011536)
Supplement: S3 Table — (DOCX) [file ppat.1011536.s003.docx]

**S3 Table.** Oligonucleotide primers used in this study.

| **Target gene** | **Primer name** | **Primer sequence (5’-3’)** |
| --- | --- | --- |
| *pgl* | *pgl*_Fwd | TCATCCTTAATTCACCCCAATC |
|  | *pgl*_Rev | CAGGTGTCCATTTACCACCA |
|  | NE202_check_F | CCTAGGGTGCCGTCTCAGCCTTGGTCTTCG |
|  | NE202_check_R | TCTGAGTTGACGCCTAATGTTGCACGAGTG |
| *gntP* | NE952_check_F | ACATCGATCATTACAGCGTTAATGCTA |
| *gntK* | NE1124_check_F | GAAGAAACAACTTGAAATGATGAAAGTG |
| *mecA* | NE1868_check_F | GGTGAAGTAGAAATGACTGAACGTC |
| Erm^r^ | Martn_ermF | TTTATGGTACCATTTCATTTTCCTGCTTTTTC |
|  | Martn_ermR | AAACTGATTTTTAGTAAACAGTTGACGATATTC |
| Kan^r^ | KanR_fwd | GACCTAGGGGTTTCAAAATCGGCTC |
|  | KanR_rev | GGCCTAGGTACTAAAACAATTCATCCAGTAAA |
| *graR* | NE481_check_F | GTTGCTGGTATTGAAGATTTCGG |
| *tarS* | NE942_check_F | CGATCAAGTGAGCGTTTAGTCAG |
| *tarM* | NE611_check_R | CAGCACCATTATTAGCATTAATATTCCTTG |
| *thrC* | NE886_check_R | GAATCGCTAAAATATCAGGTGCTTC |
| *gudB* | NE1518_check_R | CACCTAGTGCAGTTGATCTGTCG |
| *sdhC* | NE626_check_F | GCACATGTAGATTTGTTCTCAGTTGTACC |
| *leuB* | NE76_check_F | CTGTCACTGAAGGTACTGATGCCCAAGC |
| *putA* | NE239_check_R | GGTACTTATCAACTAATTCGTGGCTATCG |
| *vraG* | NE70_check_F | TGGTAACGCATGATCCTGTTGCAGCAAGC |
| *vraF* | NE645_check | CAGCTGATGTTCGTTGCCTTTGTCCACCAGAC |
| *sucC* | *sucC*_F | TACTCAAATCGCCATGCAGC |
|  | *sucC*_R | AATGACTGAAACCGTTGCCC |
| *sucA* | *sucA*_F | GGCGGTAATGGACTCGGATT |
|  | *sucA*_R | TCTACGCTATCCCCTACGTT |
| **Cloning primers** | |  |
| *pgl* | *pgl*_F | TCATCCTTAATTCACCCCAATC |
|  | *pgl*_R | CAGGTGTCCATTTACCACCA |
