# Supplementary figures and images for: Metabolic reprogramming and altered cell envelope characteristics in a pentose phosphate pathway mutant increases MRSA resistance to β-lactam antibiotics
Source: PLoS Pathog. 2023 Jul 24;19(7):e1011536. doi: 10.1371/journal.ppat.1011536 (PMC10399904; doi:10.1371/journal.ppat.1011536)

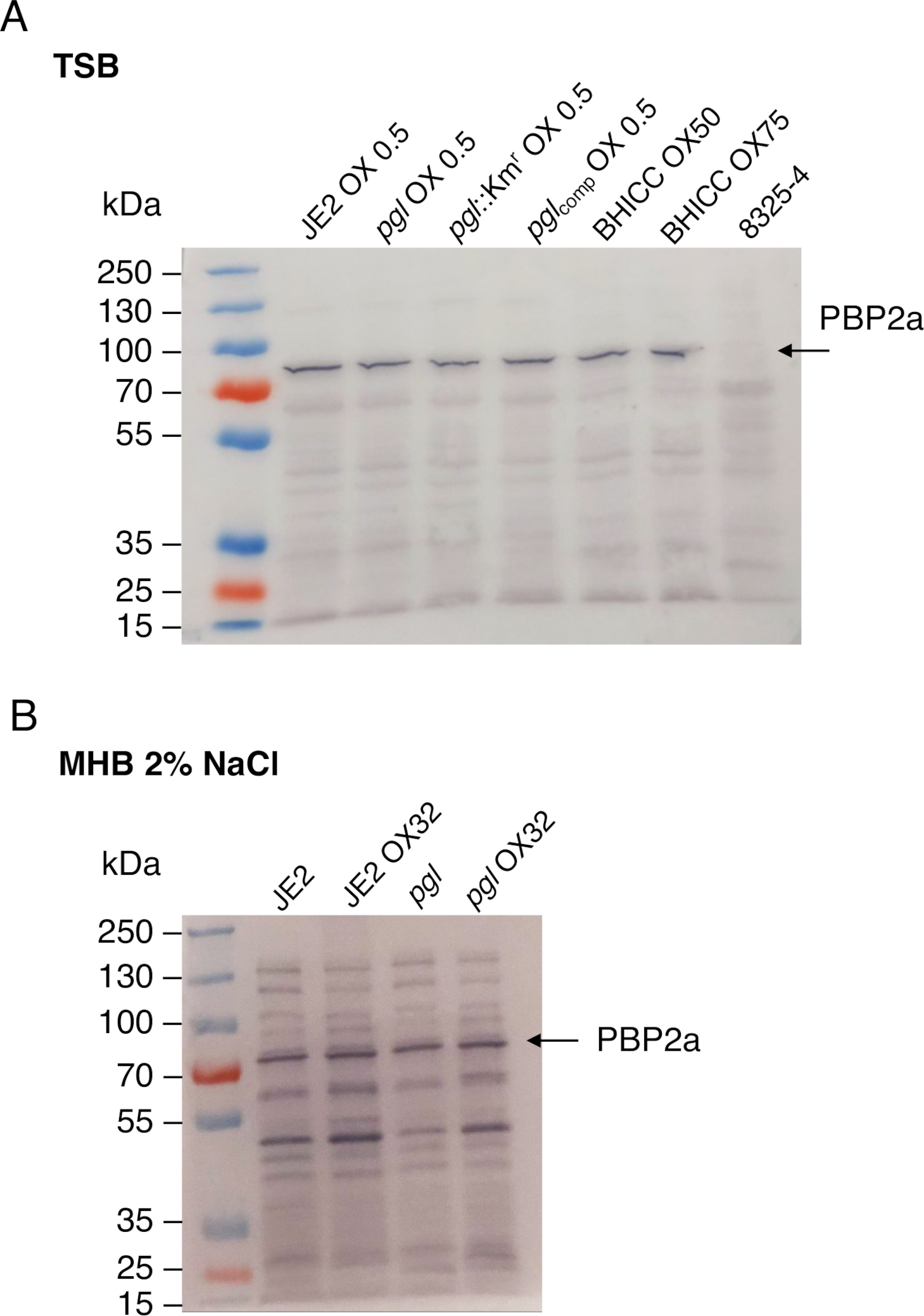

Supplement: S1 Fig — A. JE2, pgl (NE202), pgl::Kmr, and pglcomp grown for 6 h in TSB supplemented with OX 0.5 mg/ml. HoR MRSA strain BH1CC (positive control) was grown in TSB OX 50 and OX 75 mg/ml, and MSSA strain 8325–4 (negative control) was grown in TSB alone. (B) JE2 and pgl grown for 6 h in MHB 2% NaCl alone or supplemented with OX 32 mg/ml. For each sample, 8 μg total protein was run on a 7.5% Tris-Glycine gel, transferred to a PVDF membrane and probed with anti-PBP2a (1:1000), followed by HRP-conjugated protein G (1:2000) and colorimetric detection with Opti-4CN Substrate kit. Three independent experiments were performed, and representative blots are shown. (TIF) [file ppat.1011536.s004.tif]

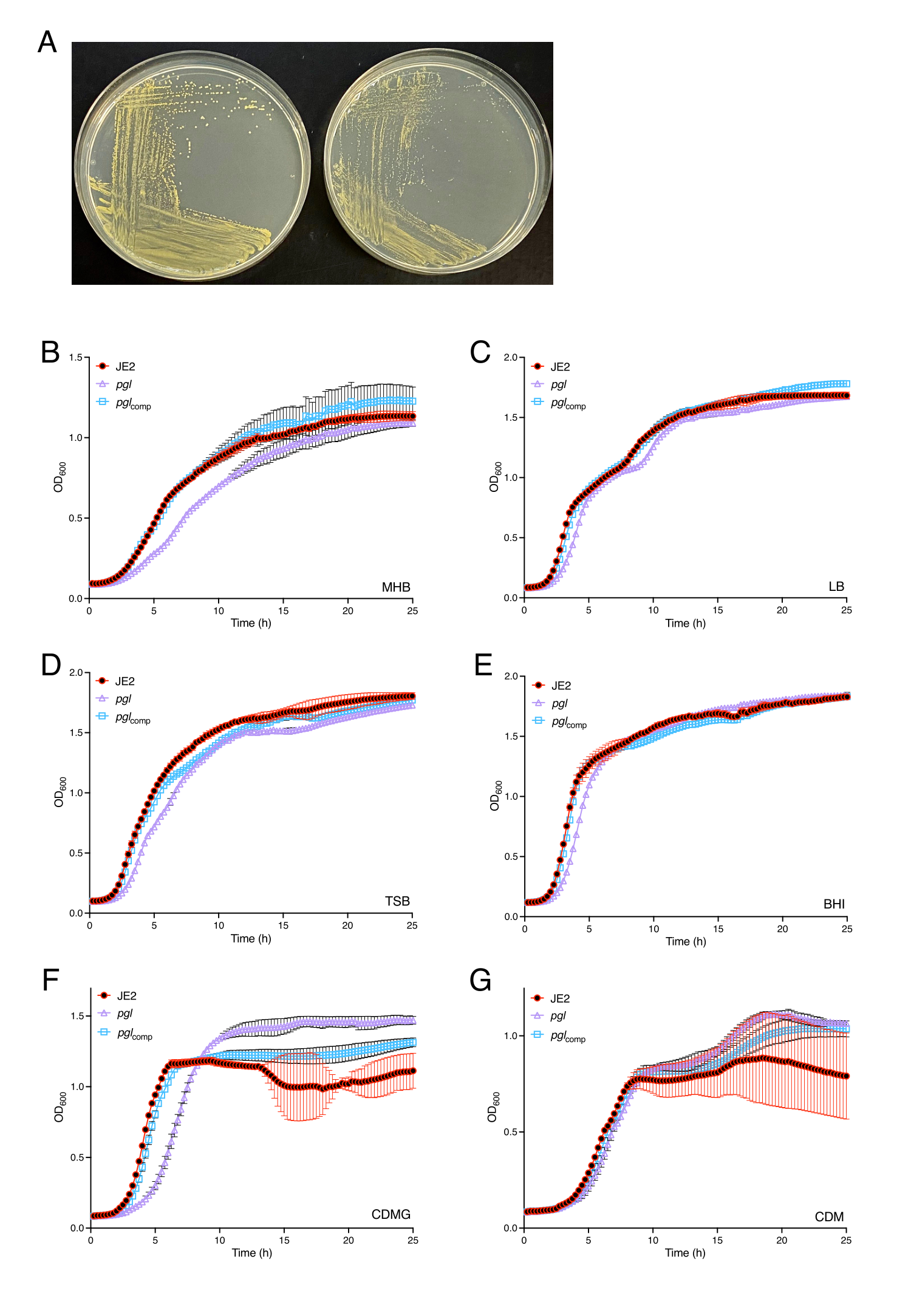

Supplement: S2 Fig — A. Isolated colonies of JE2 (left) and pgl (right) after growth on MHA for 24 h at 37°C. B-G. Growth of JE2, pgl and the complemented pgl mutant for 25 hrs at 37°C in Mueller Hinton broth, MHB (B), Luria Bertani, LB (C), Tryptic Soya broth, TSB (D), Brain Heart Infusion, BHI (E), Chemically defined media with glucose, CDMG (F) and chemically defined media with no glucose, CDM (G). Growth (OD600) was measured at 15 min intervals in a Tecan plate reader. Data are the average of 3 independent experiments plotted using GraphPad Prism V9 and error bars represent standard deviation. (TIFF) [file ppat.1011536.s005.tiff]

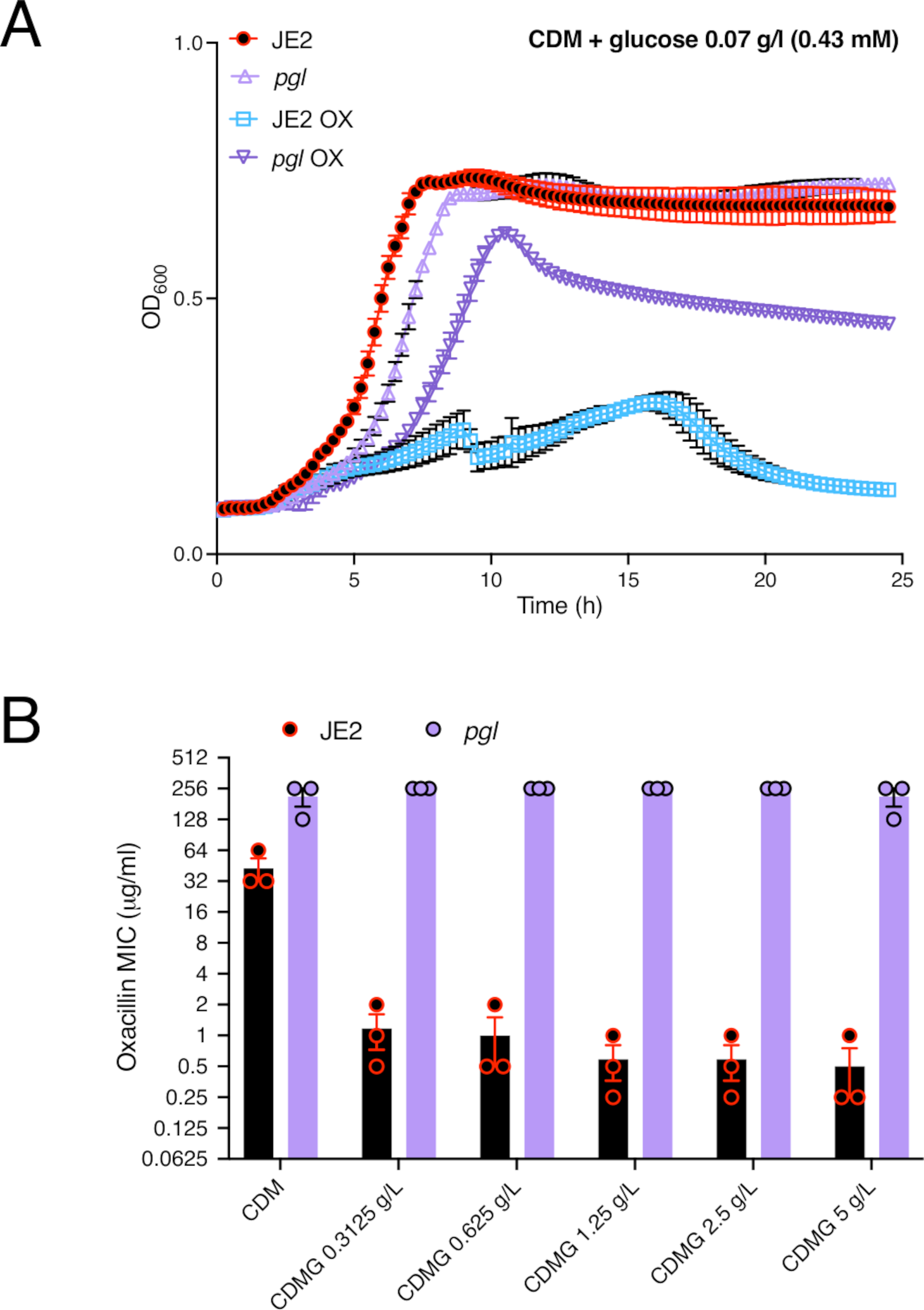

Supplement: S3 Fig — A. Growth of wild-type JE2 and pgl for 24 hrs at 37°C in chemically defined media (CDM) supplemented with 0.07 g/l (0.43 mM) glucose alone or with OX 10 mg/ml. Growth (OD600) was measured at 15 min intervals in a Tecan plate reader. Data are the average of 3 independent experiments plotted using GraphPad Prism V9 and error bars represent standard deviation. B. Oxacillin MICs of JE2 and pgl in CDM with no glucose (CDM), or CDM supplemented with glucose concentrations from 0.03–5 g/l (1.75–28 mM). Note that the Y axis (Oxacillin MIC) is a log2 scale. (TIF) [file ppat.1011536.s006.tif]

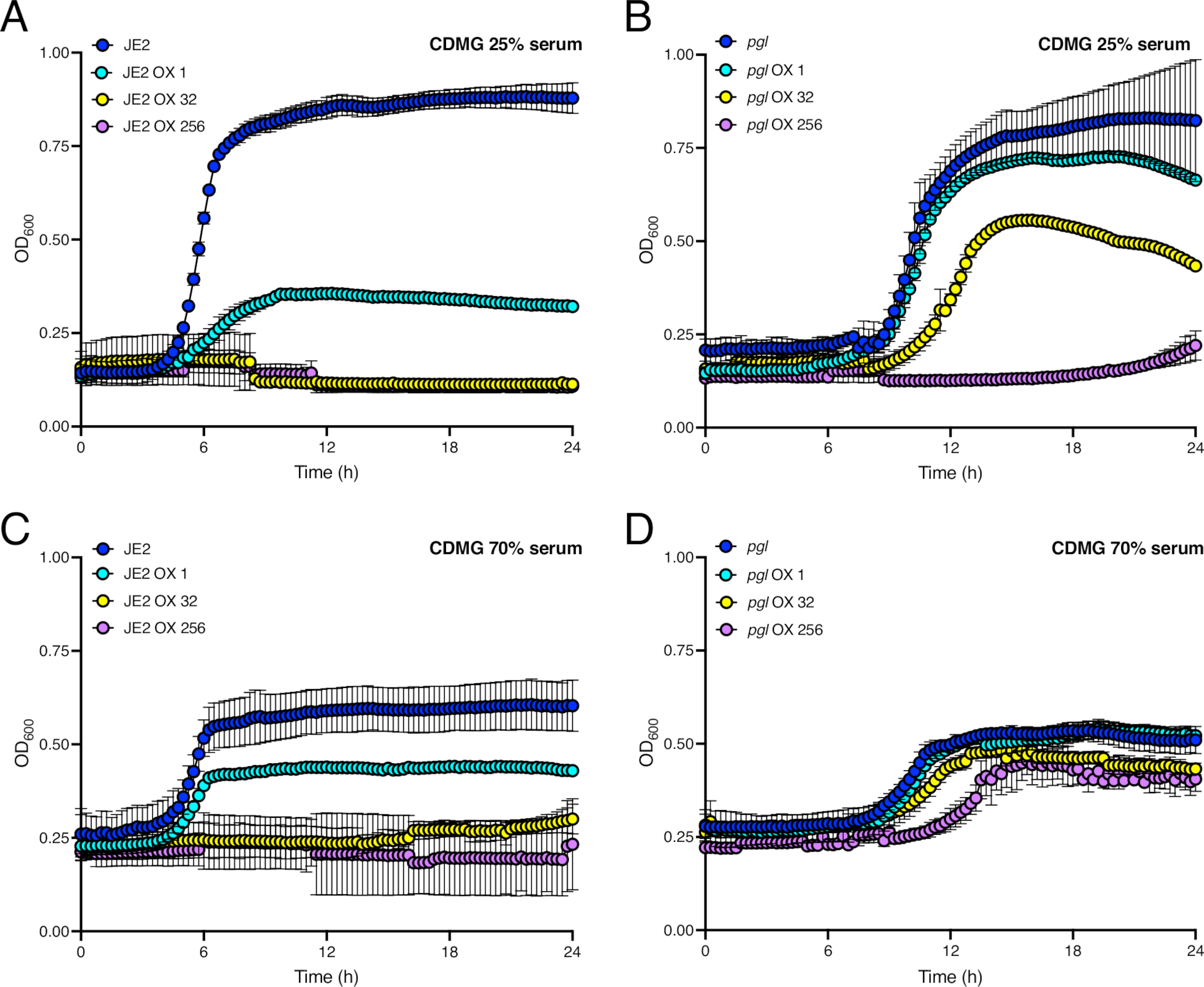

Supplement: S4 Fig — A and B. Growth of JE2 (A) and pgl (B) for 24 hrs at 35°C in CMDG 25% human serum supplemented with OX 1, 32 and 256 mg/ml. C and D. Growth of JE2 (C) and pgl (D) for 24 hrs at 35°C in CMDG 70% human serum supplemented with OX 1, 32 and 256 mg/ml. Growth (OD600) was measured at 15 min intervals in a Tecan plate reader. Data are the average of 3 independent experiments plotted using GraphPad Prism V9 and error bars represent standard deviation. (TIF) [file ppat.1011536.s007.tif]

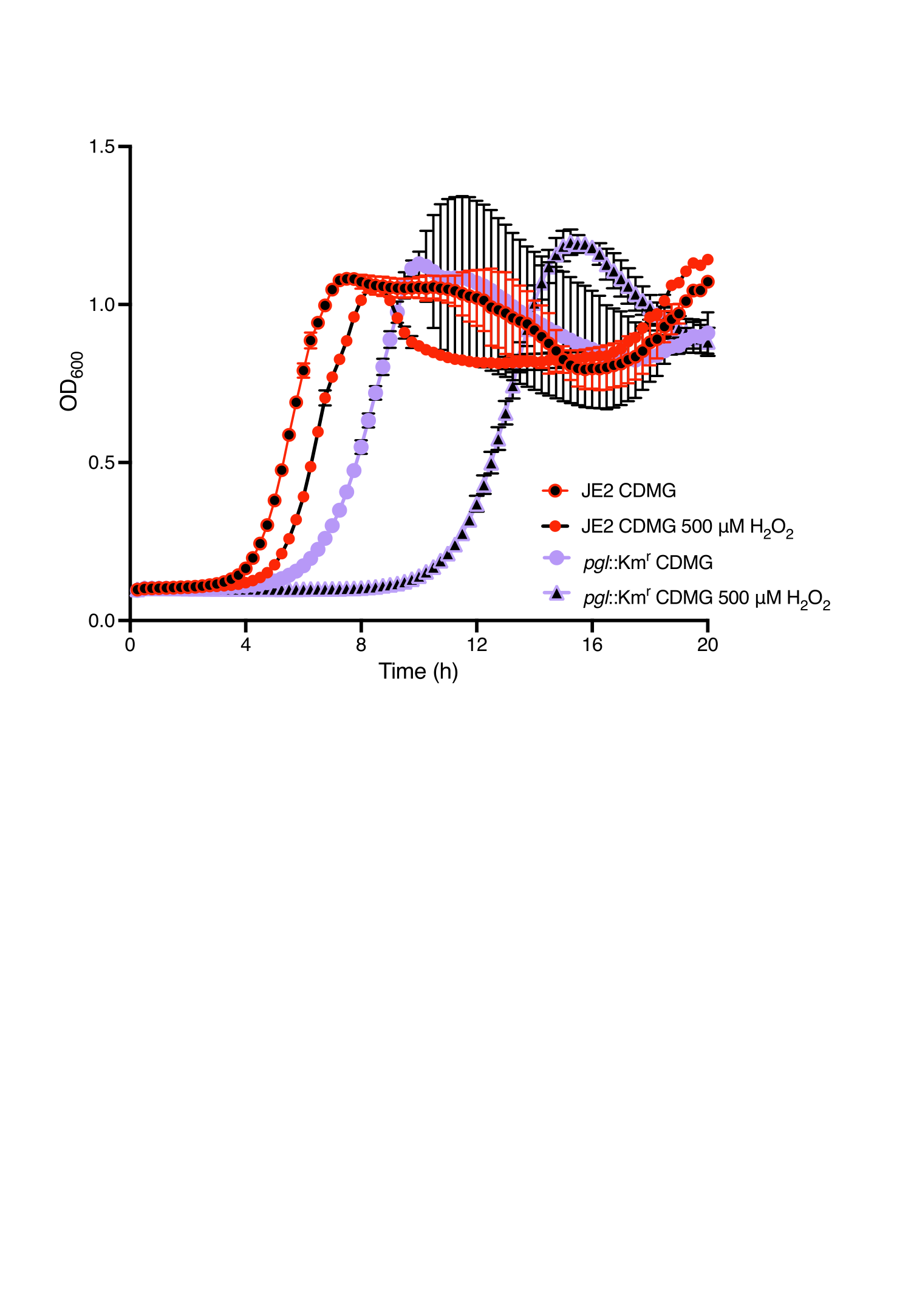

Supplement: S5 Fig — Growth of wild-type JE2 and pgl::Kmr for 24 hrs at 37°C in CDMG or CDMG supplemented with 500 mM H2O2. Growth (OD600) was measured at 15 min intervals in a Tecan plate reader. Data are the average of 3 independent experiments plotted using GraphPad Prism V9 and error bars represent standard deviation. (TIFF) [file ppat.1011536.s008.tiff]

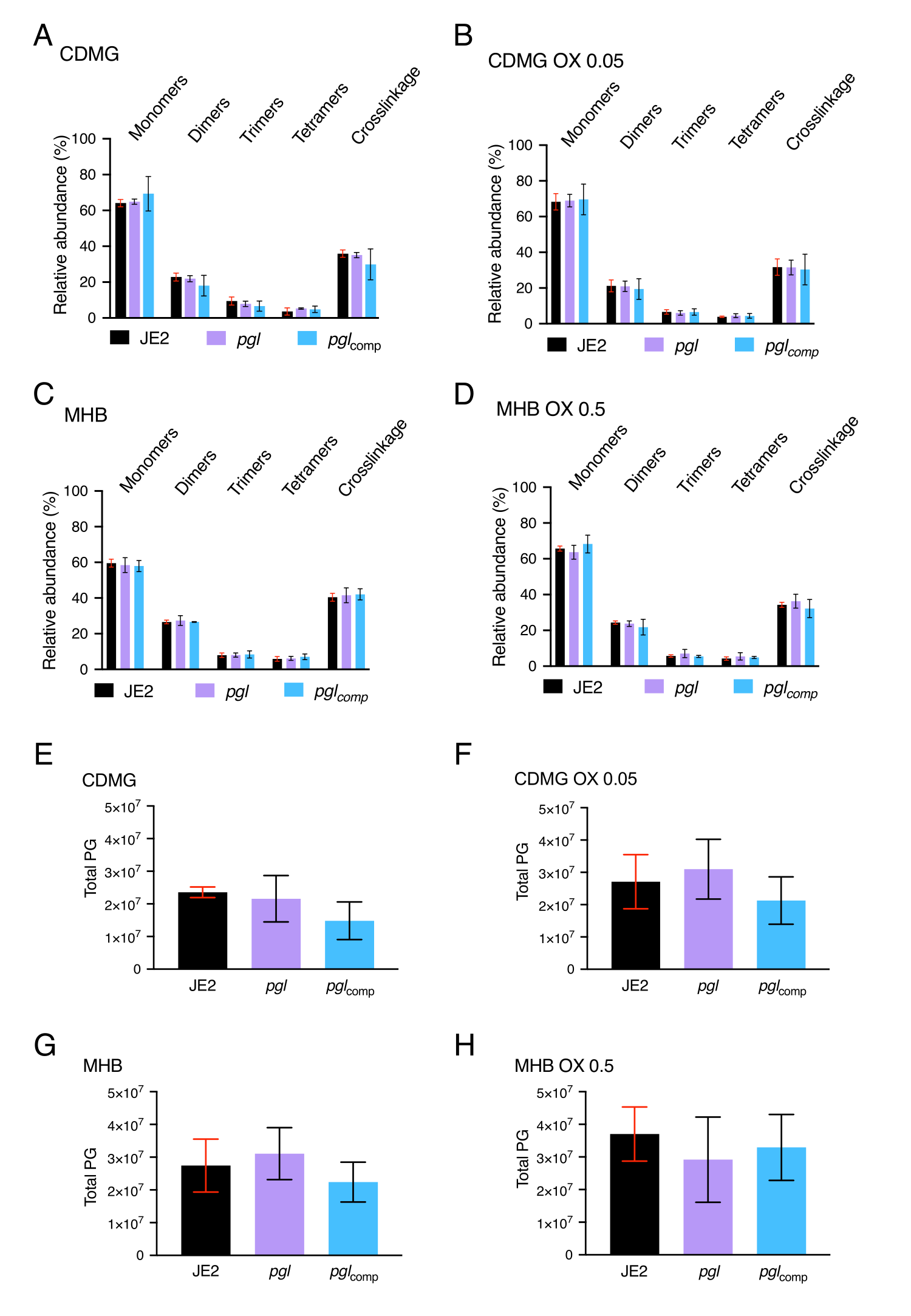

Supplement: S6 Fig — A-D. Relative proportions of cell wall muropeptide fractions based on oligomerization relative cross-linking efficiency in peptidoglycan extracted from JE2, pgl and pglcomp grown to exponential phase in CDMG (A), CDMG supplemented with OX 0.05 μg/ml (B), MHB (C) and MHB supplemented with OX 0.5 mg/ml (D). E-H. Total peptidoglycan (PG) extracted from normalised cell extracts of JE2, pgl and pglcomp grown to exponential phase in CDMG (E), CDMG supplemented with OX 0.05 μg/ml (F), MHB (G) and MHB supplemented with OX 0.5 mg/ml (H). The total PG content was calculated as the area below the chromatogram peaks/OD600 and mean and standard deviation from three/four biological repeats plotted using GraphPad Prism V9. (TIFF) [file ppat.1011536.s009.tiff]

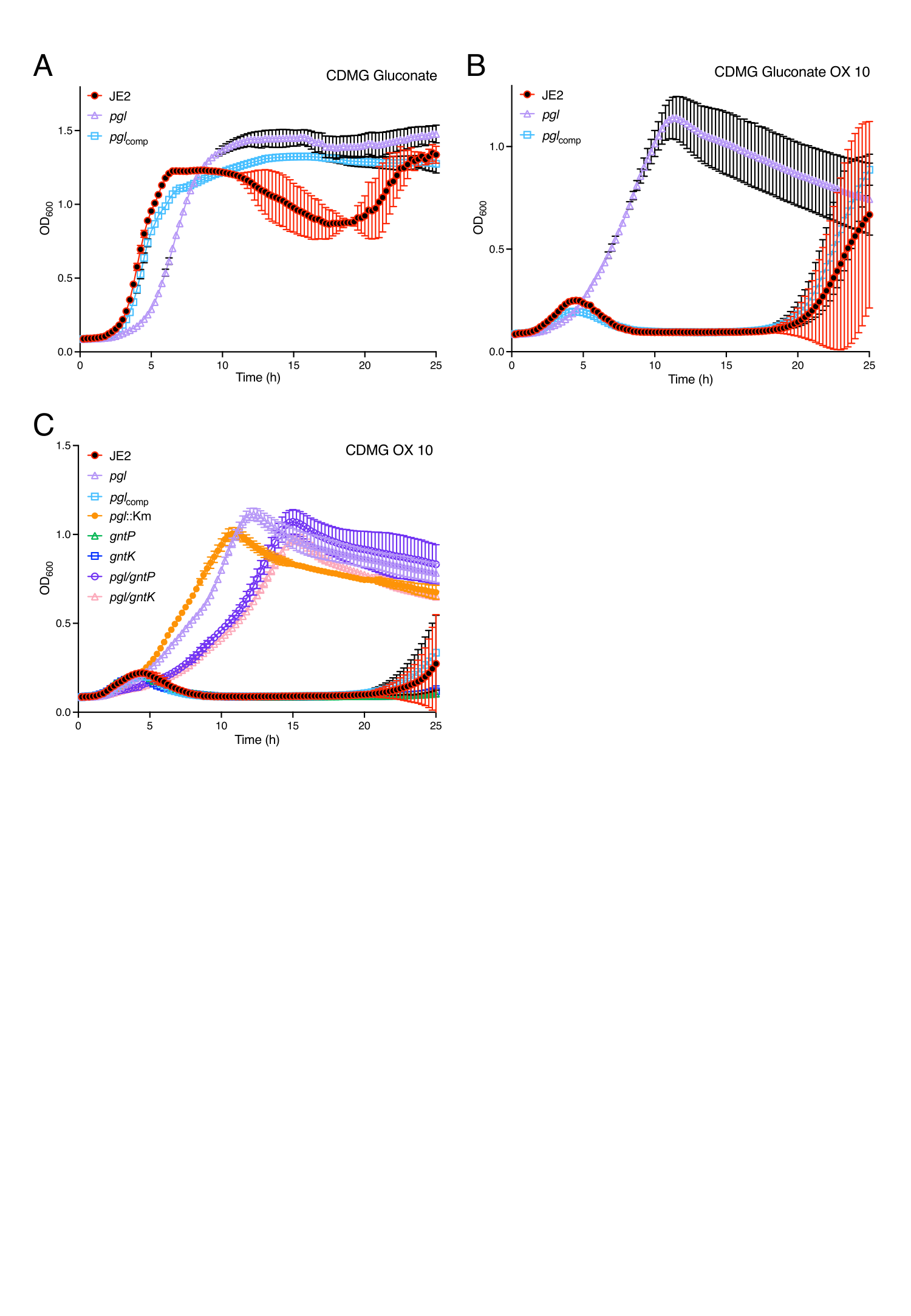

Supplement: S7 Fig — A and B. Growth of JE2, pgl and the complemented pgl mutant for 25 hrs at 35°C in CDMG supplemented with 5g/l potassium D-gluconate (0.5%) and no OX (A) or with both D-gluconate (0.5%) and OX 10 mg/ml (B). C. Growth of JE2, pgl, pglcomp pgl::Kmr, gntP (NE952), gntK (NE1124), pgl/gntP and pgl/gntK for 25 hrs at 35°C in CDMG supplemented with OX 10 mg/ml. Growth (OD600) was measured at 15 min intervals in a Tecan plate reader. Data are the average of 3 independent experiments using GraphPad Prism V9 and error bars represent standard deviation. (TIFF) [file ppat.1011536.s010.tiff]

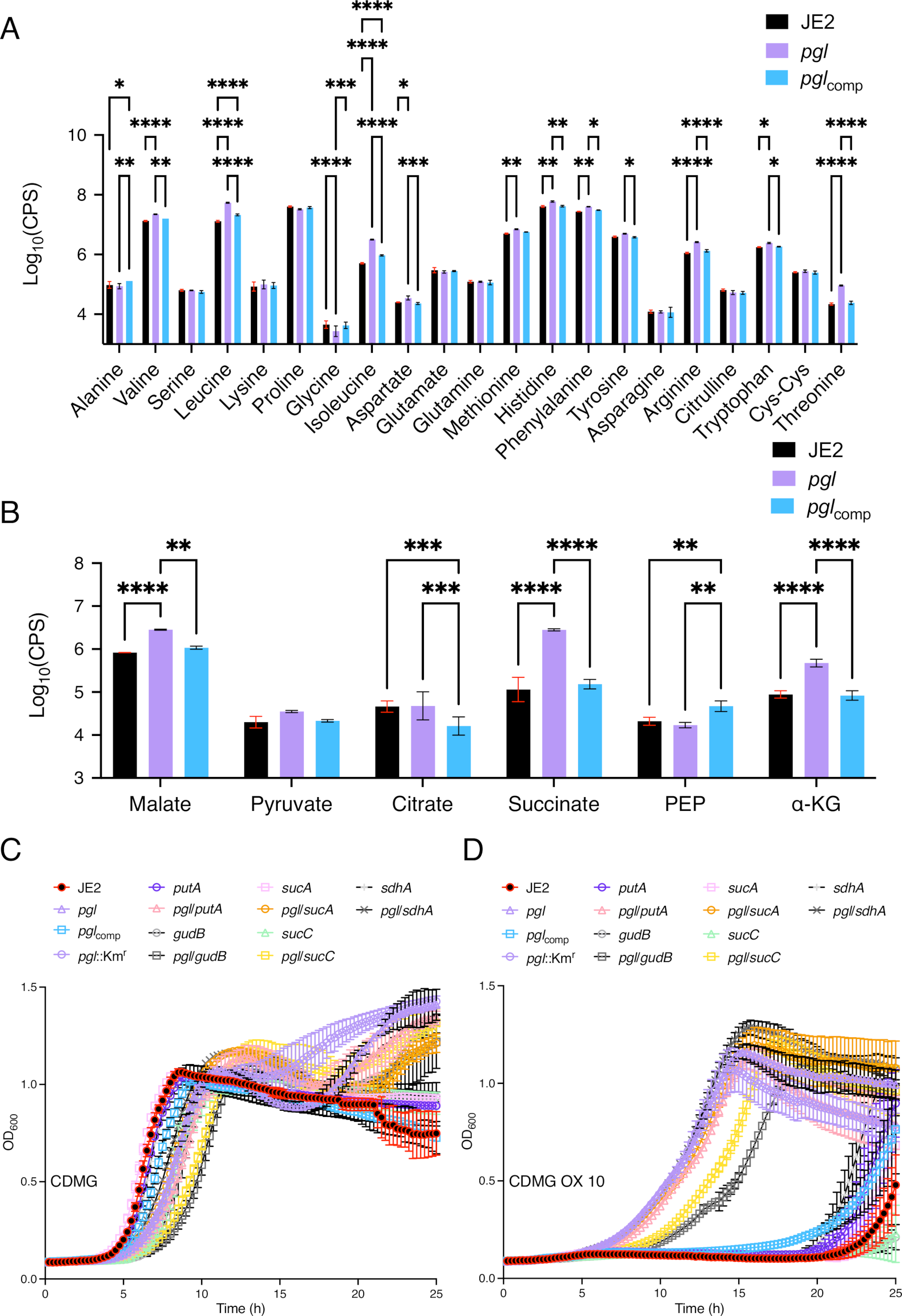

Supplement: S8 Fig — A and B. Comparison of amino acid (A) and TCA cycle metabolites (B) in supernatants of JE2, pgl and pglcomp cultures grown for 7.5 h in CDMG measured by HPLC. Cell densities (OD600) were normalized to each other before the cells were pelleted and supernatants collected. The data (CPS) shown are the average of three independent experiments and standard deviations are shown‡. Statistical significance was determined using a 2-way Anova with Dunnett’s multiple comparison test; * p<0.05, ** p<0.01, ***p<0.001, ****p<0.0001. C and D. Growth of JE2, pgl, pgl::Kmr, pglcomp, putA, gudB, sdhA, sucA, sucC, pgl/putA, pgl/gudB, pgl/sdhA, pgl/sucA and pgl/sucC for 25 hrs at 35°C in CDMG (C) and CDMG supplemented with OX 10 mg/ml (D). Growth (OD600) was measured at 15 min intervals in a Tecan plate reader. Data are the average of 3 independent experiments using GraphPad Prism V9 and error bars represent standard deviation. (TIF) [file ppat.1011536.s011.tif]

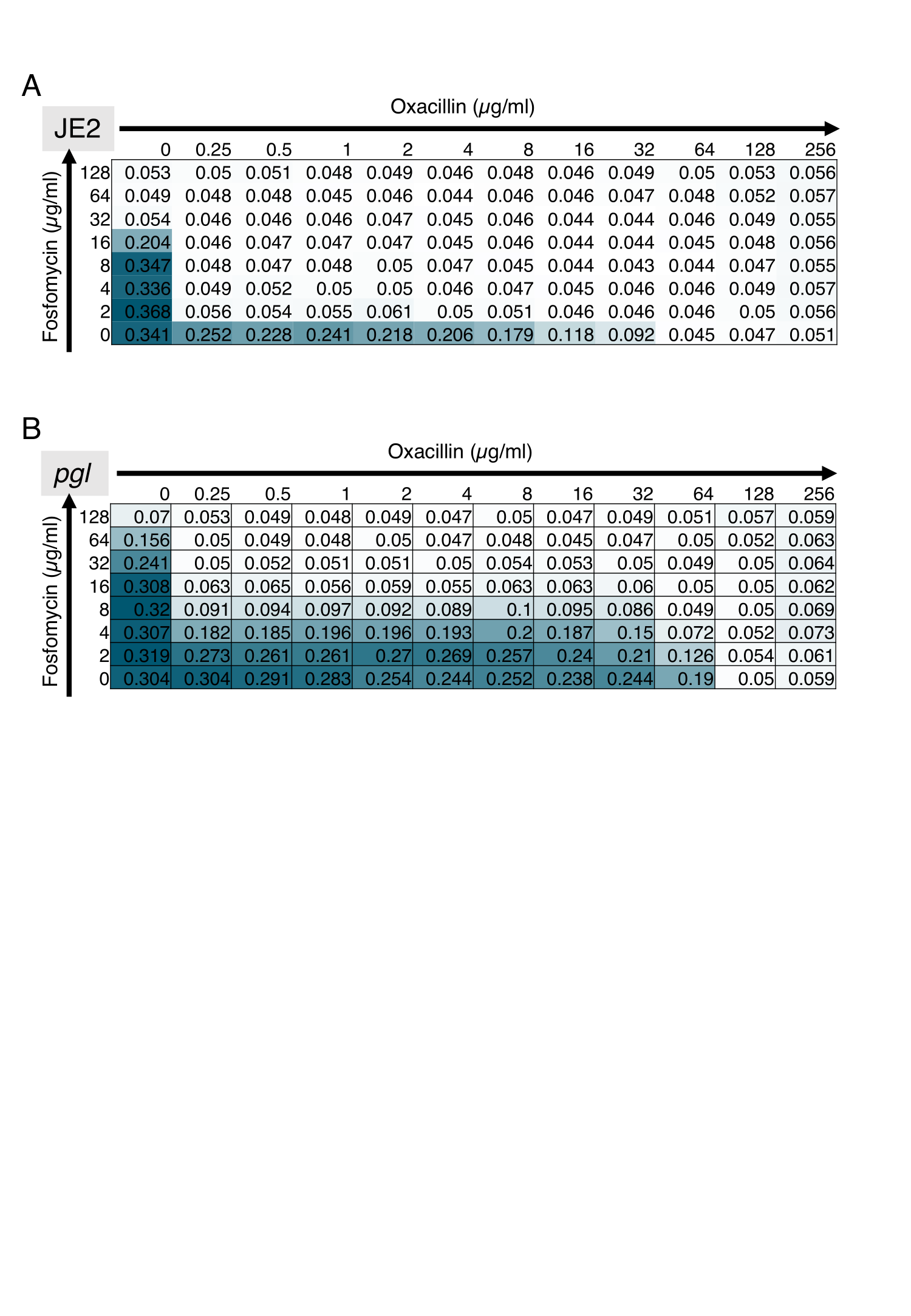

Supplement: S9 Fig — Checkerboard titration assays were conducted using fosfomycin and oxacillin with (A) JE2 and (B) pgl, grown for 24 h in Mueller Hinton 2% NaCl broth in 96-well plates. The data shown are the OD600 values for each well. The experiments were repeated at least three times and the data from a representative 96-well plate is shown. Green shaded boxes indicated wells in which significant growth was measured. (TIFF) [file ppat.1011536.s012.tiff]
