# Supplementary material for: Metabolic reprogramming and altered cell envelope characteristics in a pentose phosphate pathway mutant increases MRSA resistance to β-lactam antibiotics
Source: PLoS Pathog. 2023 Jul 24;19(7):e1011536. doi: 10.1371/journal.ppat.1011536 (PMC10399904; doi:10.1371/journal.ppat.1011536)
Supplement: Attachment — Submitted filename: Author Response PPATHOGENS-D-23-00404.pdf [file ppat.1011536.s016.pdf]

OLLSCOIL NA GAILLIMHE
UNIVERSITY OF GALWAY

Microbiology
School of Biological and Chemical Sciences

Dr Gongyi Zhang
Academic Editor
PLOS Pathogens

RE: PPATHOGENS-D-23-00404 - REVISED

08 June 2023

Dear Dr Zhang,

Please find attached our revision of PPATHOGENS-D-23-00404 "Metabolic reprogramming and altered cell envelope characteristics in a pentose phosphate pathway mutant increases MRSA resistance to β-lactam antibiotics".

We greatly appreciate the generally positive comments and constructive suggestions for improvement made by the editor and reviewers, and their work reviewing our manuscript.

In responding we have substantially revised the manuscript after careful and in-depth consideration of all of the reviewer comments. Furthermore, we report new data from 6 additional experiments suggested by the reviewers to compare:

1. PBP2a expression in the wild-type and *pgl* grown in MHB 2% NaCl media and CDMG (new Fig. S1B).
2. The impact of varying glucose concentrations on growth in OX and OX MICs (new Fig. S3A,B).
3. The impact of growth in varying human serum concentration on wild-type and *pgl* OX resistance (new Fig. S4A-D).
4. The levels of wall teichoic acids (WTAs) in the wild-type, *pgl*, pgl_{comp}, *vraG*, *pgl/vraG* and *tagO* (negative control) (new Fig. 8C).
5. The levels of lipoteichoic acids (LTAs) in the wild-type JE2, *pgl*, pgl_{comp}, *pgl*::Km^r, *vraG*, *pgl/vraG*, wild-type LAC* and *ltaS/gdpP* (negative control) (new Fig. 8D).
6. The surface charges of wild-type, *pgl*, *vraG*, *pgl/vraG* grown in CDMG and CDMG OX (new Fig. 8E).

These new data have substantially improved our manuscript and revealed that the OX resistance phenotype of *pgl* is associated with reduced LTA levels that may limit cell lysis, as well as a significantly more positive cell surface charge. VraFG/GraRS controls DltABCD-dependent D-alanylation of WTAs and LTA, which controls β-lactam resistance and surface charge, and the increased positive charge of the *pgl* mutant was partially reversed in the *pgl/vraG* double mutant. We have made a minor revision to the Title, and updated the *Abstract* and *Significance* sections to reflect these improvements.

We have included a marked-up version of the revised manuscript showing all of the revisions that have been made for the convenience of the reviewers and the editor.

Below is a point-by point response to the Reviewer's Responses to Questions:

Part I - Summary

Reviewer #1: In their manuscript entitled "Metabolic reprogramming and flux to cell envelope precursors in a pentose phosphate pathway mutant increases MRSA resistance to β-lactam antibiotics", Zeden et al investigated the metabolic and cell wall composition changes in an MRSA mutant lacking the 6-phosphogluconolactonase gene pgl that contribute to increased resistance to β-lactams. Pgl is the second enzyme in the oxidate phase of the Pentose Phosphate Pathway, and is, interestingly, the only non-essential gene in this pathway. The authors show that the pgl mutant has a higher MIC than WT for oxacillin (OX), as well as other β-lactams and bactericidal compounds that target the cell membrane. This phenotype is more pronounced in chemically defined media with glucose (CDMG) and absent in media without glucose (CDM), suggesting that carbon metabolism may have a role in the mechanism of resistance. Interestingly, the pgl mutant grows more slowly and has a reduced cell size compared to WT. Metabolomic analysis of bacteria grown in CDMG with or without OX revealed changes in key intermediates, suggesting that carbon flux was redirected from cell wall precursors. The authors hypothesize that changes to composition or architecture of the cell wall may be responsible for the antibiotic resistance phenotype. However, although the cell surface morphology is different, and the cell wall and septa between dividing cells is thicker in the mutant, the authors did not find changes to the peptidoglycan amount, cross-linking or oligomerization. The authors identify a mutant in the efflux pump VraG that reverses the resistance phenotype of the pgl mutant. Interestingly the double mutant still has slower growth kinetics and a smaller cell size. Wall teichoic acids have been shown to be required for b-lactam resistance in MRSA. Lower wheat germ agglutinin staining of the pgl mutant, compared to the WT or the double pgl/vraG mutant, showed lower levels of teichoic acids on the membrane.

This is a well written paper that includes helpful diagrams in the figures to orient the reader to the topic and specific experiments. The data is presented in a logical and easy to follow manner. Although the authors were unable to pinpoint the specific metabolic changes or the changes to the peptidoglycan that may be responsible for the increased OX resistance of the pgl mutant, the connections between the PPP and the VraFG/GraRS pathways is nevertheless novel and interesting.

Response: We are grateful to the reviewer for their work reviewing our manuscript and their positive comments on our overall findings.

Reviewer #2: The manuscript by Zeden et al. is an interesting study that seeks to understand the mechanistic underpinnings of high-level oxacillin resistance in a strain of Staphylococcus aureus lacking 6-phosphogluconolactonase, the second enzyme in the oxidative branch of the pentose phosphate pathway (PPP). S. aureus is a major human pathogen that is responsible for skin and soft tissue infections and devastating deep-seated infections. High level b-lactam resistance compounds the problem and thus, understanding the mechanisms of underlying resistance can help identify new therapeutic approaches and drug targets. The authors make the mutant (pgl) and characterize its phenotypes, noting that resistance is glucose dependent, and dependent on the alternative transpeptidase MecA (PBP2a). They rule out changes to peptidoglycan structure, TCA cycle, and amino acid metabolism, and provide evidence supporting redirected flux to cell wall precursors contributes to the resistance phenotype. Additional cell envelope changes appear to result from altered activities of teichoic acid biosynthetic enzymes. Inactivating the GraRS two-component system and VraFG ABC efflux pump re-sensitizes the pgl mutant to the b-lactam oxacillin.

The study is interesting, and the experiments are methodical in nature to unravel the mechanism. There is a great deal of attention to detail.

Response: We thank the reviewer for taking the time to review our work and their positive comments.

Reviewer #3: This manuscript by Zeden et al explores the role of the pentose phosphate pathway, specifically examining phenotypes associated with mutation of the pgl gene, in resistance to b-lactam antibiotics in MRSA.

This manuscript is well written and outlines a logical series of experiments carried out based on antibiotic MICs, and observations of cell growth kinetics and hypotheses based upon knowledge of microbial/staphylococcal metabolism.

Response: We appreciate this positive feedback from the reviewer and are grateful for their work in reviewing this manuscript.

Part II – Major Issues: Key Experiments Required for Acceptance

Reviewer #1:

1. The western blot in Figure SF1 led the authors to conclude that there are no differences in the levels of PBP2a that would explain the increased resistance of the pgl mutant. This data should be further validated. This experiment was done in TSB + OX at 6hr, when there is a slight growth difference (Fig S2D); additionally, there is no data presented about the susceptibility of the pgl strain under these conditions: if there are no differences in susceptibility in TSB then a negative result may not be informative of what is happening in CDMG. A more appropriate condition would be in CDMG OX10 at an early time point before the WT and pgl growth/survival curves diverge. Quantification of the western blot should also be presented to further validate that there is no difference in PBP2a protein expression.

Response:

PBP2a levels were also measured in JE2 and *pgl* grown in MHB 2% NaCl media. This data is now presented in new Fig. S1B of the revised manuscript. Notably, using MHB 2% NaCl, it was possible to use a higher concentration of OX (32 versus 0.5 μg/ml). As with TSB, this experiment did not reveal any significant differences in PBP2a levels between JE2 and *pgl*.

We would also reiterate that comparison of peptidoglycan structure and total amount in wild-type JE2 and *pgl* grown in CDMG or MHB 2% NaCl grown cells did reveal any changes (Fig. S4), further indicating the PBP2a expression or activity is not significantly changed in the *pgl* mutant.

Finally, in response to the reviewer's specific request, Western blot analysis of PBP2a levels in CDMG was attempted but no PBP2a was detected in any strain (see example of blot below which is not included in the paper). Further experiments will be needed to further explore this observation, which may be a technical issue or reflect a change in the PBP2a epitope targeted by the antibody.

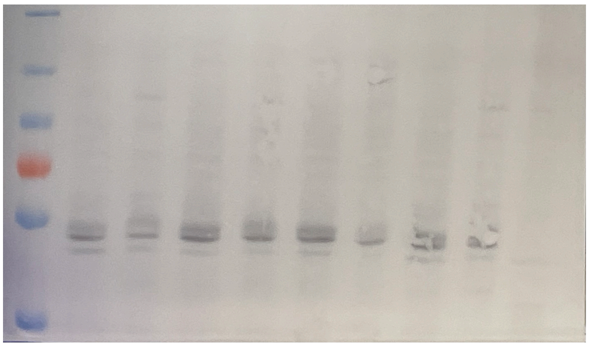

Figure. Western blot comparison of PBP2a expression in 4 biological replicates of JE2 and *pgl,* grown in CDMG OX.

Regardless, as noted in the manuscript, the increased OX resistance phenotype of the *pgl* mutant is *mecA*/PBP2a-dependent and a *pgl*/*mecA* double mutant was OX susceptible (Table 1). The *pgl*/*mecA* double mutant was also shown to be fully OX susceptible when grown in CDMG (see data below which is not included in the manuscript to limit the length of the paper)

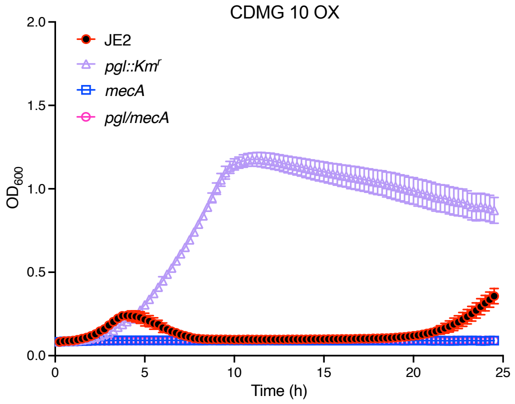

Figure. Growth of JE2, *pgl*::Km^r, *mecA* and *pgl*/*mecA* for 25 h at 35°C in CDMG supplemented with OX 10 μg/ml. Growth (OD_{600}) was measured at 15 min intervals in a Tecan plate reader. Data are the average of 3 independent experiments and error bars represent standard deviation.

2. The authors conclude that metabolic reprograming is at least partially responsible for the increased OX resistance of the pgl mutant. The experiments leading to this conclusion were done in CDMG comparing WT to the pgl mutant, where the difference in OX resistance is evident between WT and pgl. Are any of these changes also event in CDM, where both strains are susceptible to OX? If so, wouldn't that imply that these changes are not responsible for the increased resistance? In order to conclude that the metabolic reprograming is correlated to resistance, it would be important to show how metabolism is affected under conditions where the pgl mutant is susceptible to OX.

Response: We appreciate the point the reviewer is making but would emphasise that there were no conditions where both wild-type and *pgl* are OX susceptible. The *pgl* mutant was OX resistant under all conditions tested. Only the wild-type was OX susceptible in CDMG. Furthermore, comparison of wild-type JE2 and *pgl* metabolites in CDM would be very difficult to interpret given that the substrate for the PPP (glucose) is not present in the growth media and accordingly this pathway is not expected to be normally active. (Growth of *S. aureus* in CDM lacking glucose is dependent on the consumption of amino acids as carbon sources).

Our data comparing metabolism of JE2 and *pgl* in CDMG and CDMG OX, revealed metabolic reprogramming associated with the *pgl* mutation, and OX-induced changes in metabolism in both the wild-type and *pgl* strains (Fig. 5).

3. Are levels of VraFG higher in the pgl mutant in CDMG? A reasonable hypothesis would be that in the presence of exogenous glucose the pgl mutant has higher expression of this efflux pump. Confirming the level of VraF and VraG also in the absence of exogenous glucose may also help explain the resistance phenotype of the pgl/vraGF mutants grown in MHb 2% NaCl.

Response: We are very grateful for these interesting insights and suggestions. However, experiments along these lines would seem to be beyond the scope of the current manuscript, which already has a substantial amount of data. Indeed, as can be seen below, Reviewer 2 suggested that the data on VraFG/GraRS might be better suited to a separate manuscript. We decided to include the *vraG* data as it provides an insight into the mechanism of increased *pgl* OX resistance, but we accept that more work is needed to fully elucidate its role. We have added new data to show that the surface charge of *pgl* cells is significantly more positively than wild-type JE2 in CDMG OX (Fig. 8E, lines 487-489 revised manuscript). This observation is consistent with increased VraFG/GraRS-controlled DltABCD activity, which is responsible for the D-alanylation of WTAs and LTAs.

Reviewer #2: There are multiple major issues to address.

1. First, there are so many details and so much information that the manuscript is dense and difficult to digest. The model at the end does not articulate a clear explanation for the resistance phenotype. Synthesizing a better model would help.

Response: We have updated the model in Figure 9 in the revised manuscript to better illustrate the proposed differences between WT (Fig. 9B) and the *pgl* mutant (Fig. 9C), and to better depict our hypothesis for the increased OX resistance phenotype of the *pgl* mutant.

2. Second, it is not at all clear the role of the Gra-Vra complex in increased b-lactam resistance. The suppressor mutant was isolated during maker swap, so the selective pressure is unclear. Further, it is not clear how a mutation upstream of the genes results in the same phenotype as a transposon mutant.

Response:

i) As noted by the reviewer, there was no selective pressure *per se* during the experiment to swap the Erm^r marker in NE202 for a markerless deletion. As noted in the manuscript, mutation of *vraG* reverses a number

of *pgl* phenotypes, which may have conferred an advantage on *pgl*R1. Equally or perhaps more likely, this was purely serendipitous.

ii) To clarify, sequencing of the *pgl*R1 strain identified a Gln_{394}STOP substitution in VraG, and a nucleotide deletion 73bp upstream of *putA.* We did not intend to imply that the nucleotide deletion upstream of *putA* would have a phenotypic impact. Hence the possible roles of *vraG* and *putA* in the *pgl* OX phenotype were investigated by generating *pgl/vraG* and *pgl/putA* mutants. As shown in Fig. 7A,B, these experiments revealed no evidence of a role for *putA* in *pgl* OX resistance.

3. Third, the Gra-Vra experiments, while interesting, don't add to the overall model. What's the linkage between this membrane complex and the changes to the envelope? Is the activity of Gra higher in the pgl mutant? How might this happen? It seems as though this part of the story is incomplete and might be suited for a separate manuscript.

Response: We accept that questions remain about the precise role of the VraFG/GraRS multienzyme complex in the *pgl* OX resistance phenotype but decided to include this data as it provides the only insight on the mechanism of increased OX resistance in the *pgl* mutant. In the revised manuscript we have included new data showing that the *pgl* cells have a higher net positive charge (Fig. 8E), which is consistent with increased D-alanylation of WTAs and increased β-lactam resistance (new Fig. 8E). Furthermore, the activity of the Dlt enzymes that positively charge the WTAs is controlled by the VraFG/GraRS complex, and the mutation of *vraG* in the *pgl* mutant partially but significantly reversed the increased positive charge, as noted in the results (lines 487-489). We agree that further work is required to fully understand the role of VraFG/GraRS in *pgl* OX resistance and this will be the subject of a separate manuscript in the future.

Reviewer #3:

1. The role of glucose in the phenotypes observed is clear. The authors seem to have only used one concentration of glucose in their media that, in the presence of OX, induces increased cell size and lysis. I should think it informative to perform titration experiments, varying the concentration of glucose in the media, to follow the phenotypes, including growth kinetics, cell size, antibiotic resistance. This would be important given the glucose concentration (5g/L or 28 mM) employed here far exceeds physiological concentrations.

Response: New data has now been included in the revised manuscript as follows:

i. The impact of a wide range of glucose concentrations (5, 2.5, 1.25, 0.625, 0.3125, 0.15625, 0.078125 and 0 g/l) on growth of JE2 and *pgl* in CDM with and without OX 10 μg/ml has now been measured (Fig. S3A revised manuscript)
ii. The OX MICs of JE2 and *pgl* in CDMG supplemented with 5, 2.5, 1.25, 0.625, 0.3125 and 0 g/l glucose has now been measured (Fig. S3B, revised manuscript)

These new data show that even at the lowest glucose concentrations tested, wild-type JE2 growth in OX was significantly impaired. Similarly, the OX MIC of JE2 was reduced from 32-64 μg/ml to ≤1 μg/ml in glucose concentrations above 0.3 g/l, whereas the *pgl* OX MIC was 256 μg/ml in all glucose concentrations. These data indicate that the impact of glucose on MRSA OX susceptibility is important at physiologically relevant concentrations. Lines 205-209.

2. With the use of the carefully controlled experiments, along with the various mutant phenotypes, the work does add significantly more information to the role of metabolic flux in changing sensitivity to cell wall active antibiotics. Although a mechanism was not fully elucidated, the work suggests that increased carbon flux from glucose to cell envelope precursors is responsible for the observed changes in antibiotic resistance. It would add significant strength to this assumption were the exact changes in the cell envelope to have been determined. The data do imply that the impact of the pgl mutation on WTA and/or antibiotic MIC is only evident under high glucose settings. The bacteria make WTA even in TSB so experiments to more clearly identify structural alterations in WTA or LTA as a function of pgl mutation and glucose would add support to the model.

Response: New data has now been included to compare the relative levels of WTAs (Fig. 8C) and LTAs (Fig. 8D) in JE2 and *pgl* grown in CDMG and CDMG OX. Levels of WTAs were similar in all strains grown in CDMG (Fig. 8C). In CDMG OX, WTAs in the *pgl* mutant were marginally reduced in total amount compared to JE2, but ImageJ densitometry analysis from 3 independent experiments revealed that this was not significant. ImageJ densitometry analysis of LTA immunoblots revealed a significant reduction ($p<0.05$) in the relative levels of LTAs in *pgl* compared to JE2. Consistent with reduced LTA levels, the metabolomics data shown in Fig. 5 showed that levels of UDP-Glucose are significantly decreased in *pgl* compared to JE2 with or without oxacillin indicative of defective glycolipid production.

The reduced levels of TAs in *pgl* correlated with the reduction in WGA binding (Fig. 8A) and the increased susceptibility of the *pgl* mutant to Congo red (Fig. 8B), which targets LtaS. Furthermore, as LTA accumulates at the site of cell division (1), reduced levels of this glycopolymer may contribute to the cell division and cell size differences evident in *pgl* (Fig. 6).

Hesser et al (2) recently proposed that the production of long and abundant LTAs in *S. aureus* promotes cell lysis, whereas abundant WTA levels have the opposite effect and limit cell lysis. Our data appear to be consistent with this hypothesis and revealed a correlation between significantly reduced LTA levels in *pgl* and reduced cell lysis under OX stress. In turn, the reduced cell lysis of *pgl* may also contribute to increased OX resistance.

These data also raise questions about the impact of the altered WTA:LTA ratio on the *pgl* cell envelope and/or the post-translational modification(s) of teichoic acids. Analysis of *tarS/pgl* and *tarM/pgl* mutants revealed that WTA glycosylation was not required for *pgl* OX resistance (Fig. S11). Because TAs play an important role in the overall cell surface charge, a cytochrome c binding assay was performed on JE2 and *pgl* grown in CDMG and CDMG OX. Interestingly *pgl* cells were significantly more positively charged than JE2 cells in CDMG OX (Fig. 8E). A similar trend was observed in CDMG, but did not reach significance. Consistent with published data, VraG is required for the net positive charge of the cell and the *vraG* mutant was more negatively charged than wild-type, albeit not significantly (Fig. 8E). The *pgl/vraG* mutant exhibited an intermediate phenotype compared to JE2 and *pgl*, indicating that the *vraG* mutation partially restored a wild-type surface charge in the *pgl* mutant, which is consistent with the restoration of wild-type OX susceptibility. The increased positive charge and drastic reduction in LTAs are indicative of a significant alterations in the *pgl* cell envelope. Moreover, these observations are consistent with increased VraFG/GraRS-dependent, Dlt-mediated D-alanylation of TAs and increased OX resistance in *pgl*.

We have reworked the final two sections of the Results (lines 460-503) and relevant parts of the Discussion (lines 592-622).

Separately, and consistent with previously published data, the *vraG* mutation alone or in combination with *pgl*, was associated with significantly increased susceptibility to polymyxin B (Table 1, lines 426-428).

3. The experiments presented here show that under some culture conditions glucose and pgl mutation can affect antibiotic MIC and cell ultrastructure. Of interest to the readers of PLOS Pathogens would be to know how these changes affect in vivo pathogenesis. Even using a relevant in vivo substitute in in vitro experiments, like growth phenotypes/MICs in human serum (where the glucose concentration is lower) would be informative. Are there any in vivo

phenotypes associated with the pgl mutants, in MRSA or MSSA, with or without the presence of antibiotic?

Response: We have now generated new data to compare the growth of wild-type JE2 and *pgl* in CDMG supplemented increasing concentrations of human serum (10, 25, 50 and 70% serum). The growth data JE2 and *pgl* in 25% serum/75% CDMG and 70% serum/30% CDMG are now included in new Fig. S4A-D of the revised manuscript. These new data show that the wild-type cannot grow at OX concentrations > 1 μg/ml whereas the *pgl* mutant grew at OX concentrations up to and including 256 μg/ml.

Furthermore, we have also now measured the OX MICs of JE2 and *pgl* mutant in MHB and CDMG supplemented with a range of human serum concentrations. This data is presented in new Table S1 of the revised manuscript and shows that the *pgl* mutant was significantly more resistant to OX than JE2 in all serum concentrations.

Lines 208-215.

Part III – Minor Issues: Editorial and Data Presentation Modifications
Please use this section for editorial suggestions as well as relatively minor modifications of existing data that would enhance clarity.

Reviewer #1:

1. Lines 263/264 are confusing: if I understand correctly, the "growth… was similar" statement refers to growth with or without gluconate; as written, the sentence implies that the growth was similar between gluconate alone and OX.

> **Response:** This sentence has been rephrased as follows:
> Line 268-270: "Growth and OX resistance of wild-type JE2, *pgl* and *pgl*$_{comp}$ were similar in CDMG supplemented with 5 g/l D-gluconate and CDMG OX D-gluconate (Fig. S7A, B)."

2. The resistance phenotype of pgl/gntP and pgl/gntK double mutants is taken as evidence that the gluconate shunt genes do not play a role in the pgl OX resistance phenotype. However, in Fig S5C, the double mutants do show a growth delay in the presence of OX, rather than phenocopying the pgl mutant alone. This suggests that the gluconate shunt genes have a partial role, so the conclusion of this experiment, as stated, is too strong.

Response: We accept that mutation of the gluconate shunt genes in the *pgl* mutant was accompanied by a growth delay. However, the *pgl/gntP* and *pgl/gntK* mutants reached the same cell density in CDMG OX as *pgl* (now Fig. S7C in the revised manuscript). To better reflect these data, we have revised the text of the results as follows:

Lines 261-262: "**Exogenous D-gluconate or mutation of the *gntPK* gluconate shunt genes did not restore wild-type OX resistance in the *pgl* mutant.**"

Lines 270-272: "Inactivation of *gntP* or *gntK* in the *pgl* mutant was accompanied by a modest growth delay in CDMG OX but did not restore wild-type levels of OX susceptibility (Fig. S7C). Therefore, exogenous D-gluconate and the gluconate shunt genes are not significantly involved in the increased OX resistance phenotype of the *pgl* mutant."

Legend for Fig. S7:
"Fig. S7. Exogenous addition of D-gluconate or mutation of the gluconate shunt genes *gntP* or *gntK* did not restore wild-type OX resistance in the *pgl* mutant"

3. Statistics should be added to Fig S6A/B since the differences between the conditions are difficult to judge by eye. For panel A/threonine and panel B/a-KG, where there no measurements for the JE2 and pglcomp strains or are these values 0?

Response: Statistical analysis (2-way Anova with Dunnett's multiple comparison test) has now been performed on the data in what is now Fig. S8A and Fig. S8B of the revised manuscript.
The peaks for threonine and a-KG were below the signal-to-noise (S/N) threshold and thus filtered out. We have now lowered the S/N threshold and integrated the corresponding peaks. This has allowed us to also perform statistical analysis on these metabolites in JE2, *pgl* and pgl_{comp}. Fig. S8A has been revised to show this.

4. Lines 417/418 refer to the growth of the pgl/graR mutant in CDMG OX in Figure 7B. However, this panel does not show that particular mutant.

Response: We thank the reviewer for spotting this omission. The growth data for the *graR* and *pgl/graR* mutants has now been included in revised Fig. 7A and B, and the figure legend updated accordingly.

5. I could not find the time point of the metabolomic analysis presented in Figure 5 either in the figure legend or in the materials and methods section. Please include this information.

Response: This information was provided in the methods (line 895 revised manuscript) and for additional clarity is now also included in the legend for Figure 5 as follows"

"**Fig. 5. Heatmap comparison of cell wall, pentose phosphate pathway (PPP)/glycolysis, TCA cycle, redox, nucleotides and amino acid metabolites in JE2, *pgl* and *pgl*$_{comp}$.** Whole cell metabolomics was performed on JE2, *pgl* and *pgl*$_{comp}$ grown in CDMG and CDMG OX 10 mg/ml and the cells collected after 4-5 hours (early exponential phase). ….."

6. Can the authors comment in the discussion on what the potential mechanism for decreased cell size and increased cell wall thickness may be? Given that the pgl/vraG mutant does not reverse that phenotype, but does reverse the resistance phenotype, one would conclude that these changes to the cell wall are not in themselves sufficient for β-lactam resistance. Can the authors comment on the relationship between these two phenotypes?

Response: To address this we have added the following revised sentence to the discussion of the revised manuscript: "In contrast to its effect on OX resistance, mutation of *vraG* did not reverse the impact of the *pgl* mutation on LTA levels or cell size suggesting that the broader metabolic consequences of the disrupted PPP on cell size are distinct from the more precise VraFG/GraRS-dependent increase in OX resistance." Lines 611-614 revised manuscript.

7. For Figure 9, panel B: it would be helpful to lay out the model contrasting WT vs pgl in two separate diagrams. As drawn, it's hard to follow what the authors propose is happening in the pgl mutant that is different from the WT strain.

Response: We have updated the model in Figure 9 to better illustrate the proposed differences between WT (Fig. 9B) and the *pgl* mutant (Fig. 9C). (Reviewers 2 and 3 also requested an improved model).

8. It would be great if the authors could highlight in more depth the glucose-dependent antibiotic resistance of the pgl mutant and its relationship to clinical antibiotic testing. The authors touch on this in the discussion (lines 511-515), but given the significant implications of this, another sentence or two would be beneficial.

Response: We have added new data showing that oxacillin resistance in JE2 is dependent on physiologically relevant glucose concentrations (Fig. S3, revised manuscript).

Reviewer #2: Additional minor issues include:

1. Line 210: do you mean glucose-dependent? Please rephrase

Response: Rephrased as suggested (lines 192 and 210 revised manuscript)

2. Line 225: it's significant or not. Do you mean more pronounced or exaggerated? Please rephrase

Response: We have rephrased this as follows: "The significant OX-induced increase in MRSA cell size, which we and others have previously reported (3-7), was more pronounced in wild-type JE2 and *pgl*$_{comp}$ than the *pgl* mutant (Fig. 3C)." Lines 229-231 revised manuscript.

3. Line 307: use of the leuB mutant needs better justification.

Response: On further reflection, we accept that the *leuB* data is not significantly adding to the conclusions of the paper, and it has been removed from the revised manuscript. Fig. S8C and D have been revised accordingly. Lines 312-315 revised manuscript.

4. Fig 7: It's hard to see the strains and evaluate the data. Perhaps a bar graph with final OD values would be better here and in similar figures.

Response: We acknowledge the validity of this point by the reviewer, but would point out that the graphs, as presented reveal patterns of growth that are not captured by final OD values. An example of this is the growth of the *pgl*R1 strain compared to wild-type JE2 in CDMG, as noted by the reviewer in point 6 below. In this context, we would hope that the reviewer will accept our decision not to change the presentation of the data in Figure 7.

5. Line 409: please delete the repeated reference to the figure

Response: Corrected as requested.

6. Lines 456-458: Is this really restored to WT levels? Statistical analysis can help here.

Response: As noted in this sentence, we were referring particularly to growth in CDMG OX. To emphasize this, we have revised that sentence to include the word "particularly" as follows: "…..a *pgl* markerless transposon mutant that had reverted to wild-type patterns of growth, particularly in CDMG OX, was isolated (Fig. 7A, B)." Lines 410-412 revised manuscript

7. Please provide a reference for the CDM formulation

Response: Reference to Halsey et al paper now included (line 683 revised manuscript).

8. LB is lysogeny broth, not Luria Broth. See reference:

Response: We have changed this to lysogeny broth/Luria Bertani broth (lines 678-679 revised manuscript). We have retained reference to Luria Bertani broth because for many researchers LB has come to colloquially mean Luria Bertani broth.

9. A better description of how the cells were grown is required to reproduce the experiments elsewhere, especially since the extent of aeration can affect metabolism. With shaking? What was the vessel-medium ratio?

Response: All growth data presented in the figures of this manuscript was generated in a Tecan plate reader, details of which are already provided in the methods section (Lines 723-742 revised manuscript).

10. References beyond 92 are missing, yet are cited in the supplementary table (for instance ref 96)

Response: We thank the reviewer for noting this error associated with the EndNote references for Table S1. This has been corrected in the revised manuscript, in which Table S1 now has its own references.

Reviewer #3:

1. y-axis on fig 2A is not scaled properly.

Response: These data are presented this way for illustrative purposes and we have now noted in the Fig. 2A legend of the revised manuscript that Note that the Y axis (Oxacillin MIC) is a log2 scale. This was also noted in the legends of new Figure S3 and Figure S11, which have MIC data presented in the same way.

2. Fig. 9A can be made more clear? For instance, what is shown in the division septum in the top right? Why would it not be present in the pgl mutant depicted below?

Response: We have updated the model in Figure 9 to better illustrate the proposed differences between WT (Fig. 9B) and the *pgl* mutant (Fig. 9C). (Reviewers 1 and 2 also requested an improved model). The top right

panel (Fig 9A) illustrates the lysis of JE2 wild-type cells in CDMG OX which does not happen in *pgl* cells grown in the same condition.

Sincerely,

Merve S. Zeden and James P. O'Gara

References

1. **Reichmann NT, Picarra Cassona C, Monteiro JM, Bottomley AL, Corrigan RM, Foster SJ, Pinho MG, Grundling A.** 2014. Differential localization of LTA synthesis proteins and their interaction with the cell division machinery in Staphylococcus aureus. Mol Microbiol **92:**273-286.
2. **Hesser AR, Matano LM, Vickery CR, Wood BM, Santiago AG, Morris HG, Do T, Losick R, Walker S.** 2020. The length of lipoteichoic acid polymers controls Staphylococcus aureus cell size and envelope integrity. J Bacteriol **202.**
3. **Salamaga B, Kong L, Pasquina-Lemonche L, Lafage L, von Und Zur Muhlen M, Gibson JF, Grybchuk D, Tooke AK, Panchal V, Culp EJ, Tatham E, O'Kane ME, Catley TE, Renshaw SA, Wright GD, Plevka P, Bullough PA, Han A, Hobbs JK, Foster SJ.** 2021. Demonstration of the role of cell wall homeostasis in Staphylococcus aureus growth and the action of bactericidal antibiotics. Proc Natl Acad Sci U S A **118.**
4. **Thalso-Madsen I, Torrubia FR, Xu L, Petersen A, Jensen C, Frees D.** 2019. The Sle1 Cell Wall Amidase Is Essential for beta-Lactam Resistance in Community-Acquired Methicillin-Resistant Staphylococcus aureus USA300. Antimicrob Agents Chemother **64.**
5. **Lorian V.** 1985. Low concentrations of antibiotics. J Antimicrob Chemother **15 Suppl A:**15-26.
6. **Pinho MG, Errington J.** 2003. Dispersed mode of Staphylococcus aureus cell wall synthesis in the absence of the division machinery. Mol Microbiol **50:**871-881.
7. **Nolan AC, Zeden MS, Kviatkovski I, Campbell C, Urwin L, Corrigan RM, Grundling A, O'Gara JP.** 2022. Purine Nucleosides Interfere with c-di-AMP Levels and Act as Adjuvants To Re-Sensitize MRSA To beta-Lactam Antibiotics. mBio doi:10.1128/mbio.02478-22**:**e0247822.
